# Selected food items adulteration, their impacts on public health, and detection methods: A review

**DOI:** 10.1002/fsn3.3732

**Published:** 2023-10-05

**Authors:** Abdulmajid Haji, Kasahun Desalegn, Hayat Hassen

**Affiliations:** ^1^ Department of Post‐Harvest Management College of Agriculture and Veterinary Medicine, Jimma University Jimma Ethiopia

**Keywords:** adulteration, detection, food adulteration, food items, public health

## Abstract

Every living thing requires food to survive. Clean, fresh, and healthy foods are important to human health. Today, food is affected by various counterfeits. Adulteration of food is the intentional deterioration of the quality of food offered for sale by either the addition or substitution of an inferior substance or by the omission of a valuable ingredient. Economically motivated adulteration is the intentional adulteration of food for financial gain, and has enormous public health implications, making it an important issue in food science. Almost every food, including milk and dairy products, fats and oils, fruits and vegetables, grain foods, coffee, tea, honey, etc., is susceptible to adulteration. It is difficult to find food that is free from adulteration. Consumption of adulterated food contributes to numerous diseases in society, ranging from mild to life threatening. Therefore, detection of adulteration in food is essential to ensure the safety of the food we consume. To provide consumers with food that is free of adulterants, various detection methods such as physical, chemical, biochemical, and molecular techniques are used to identify adulterants in food. This review aims to provide up‐to‐date information on food adulteration, its impact on health, and the analytical techniques used to detect adulteration in food.

## Graphical abstract

Every living thing needs food to survive. Clean, fresh, and healthy food is the most important for human health. Today, however, food is affected by various counterfeits. Adulteration of food is the intentional deterioration of the quality of food offered for sale, either by the addition or substitution of an inferior substance or by the omission of a valuable ingredient.

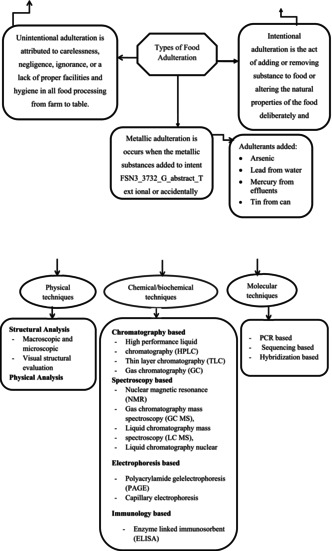

## 1 INTRODUCTION

Food is an important lifestyle issue and is defined as any substance consisting of carbohydrates, water, fats, and proteins that can be consumed by humans and animals for nutrition (Choudhary et al., 2020). It is a basic requirement for vital nutrients and contributes to human growth and maintenance. In addition, the consumption of quality foods plays a critical role in providing nutrients necessary for maintaining good health, full growth, and development of the body (Goyal et al., 2022; Tomar & Alka, 2022). Health is wealth; therefore, people must take care to maintain good health. It is not only the quantity but also the quality of food, which is key to leading a healthy life (Koul et al., 2020). However, adulteration compromises food quality (Choudhary et al., 2020; Rath, 2022).

Food adulteration is widespread global food fraud that endangers food safety (Meghwal et al., 2022). Food adulteration can be done deliberately by degrading the quality of food offered for sale by adding or substituting low‐quality materials or removing valuable ingredients from essential foods (Bansal et al., 2017). These evil and reckless methods of massive wealth acquisition pose a significant threat to human life (Ghosh et al., 2022). The deliberate adulteration of food for financial gain is known as economically motivated adulteration (EMA) (Everstine et al., 2013). It is not only an economic crime, but it also poses a significant threat to public health (Spink & Moyer, 2011). Various food items are exposed to food adulteration, including dairy products, grains, seafood, oils, alcoholic drinks, honey, fruits, and vegetables, and are adulterated in many ways (Dwivedi, 2022; Essuman et al., 2022; Ghosh et al., 2022; Nagvanshi, 2015; Tomar & Alka, 2022). Thus, it is difficult to find food that is free from one adulterant. The latest data on food items show that nearly 50% of the food consumed every day is adulterated (Koul et al., 2020). According to another report, around 22% of foods are adulterated each year (Chachan et al., 2021; Koul et al., 2020; Thangaraju et al., 2021).

Food can be adulterated deliberately or unintentionally. Deliberate adulteration of food is usually performed for financial reasons and during processing. Accidental adulteration is usually caused by ignorance, negligence, or a lack of control (Ayza & Belete, 2015). Metallic contaminants can also enter food through environmental pollution or during the food manufacturing process and can be present in trace amounts in food (Banti, 2020). The biggest challenges of food adulteration are public health issues, lack of recognition in the market due to low originality, and decline in consumer confidence (Anita & Neetu, 2013).

Food adulteration is a common problem in both developed and developing countries. A less‐developed country is even more vulnerable to food adulteration problems (Hossain et al., 2008) owing to lack of strong regulatory systems and policies (Ayza & Belete, 2015). Food adulteration is widespread in many developing countries, including India, China, Ethiopia, Mexico, Pakistan, Bangladesh, Vietnam, Indonesia, Afghanistan, and Somalia (Chachan et al., 2021; Pal & Mahinder, 2020). It is a serious problem that can have several negative consequences for consumers, including health risks, economic losses, and social unrest (Wu et al., 2017). Adulterated foods are responsible for mild‐to‐severe health effects. Diarrhea, nausea, allergic reactions, diabetes, and cardiovascular disease are often observed upon consumption of adulterated food (Momtaz et al., 2023). Approximately 57% of people develop health problems due to the ingestion of adulterants and contaminants (Choudhary et al., 2020; Pal & Mahinder, 2020; Thangaraju et al., 2021; Islam et al., 2022).

Food adulteration can also cause financial damage. When food is adulterated, consumers lose confidence in food safety and quality, which reduces demand and sales (Ayza & Belete, 2015). This can harm the reputation and profitability of food businesses, leading to job losses and reduced economic growth (Banti, 2020; Chekol et al., 2022; Islam et al., 2022). Food adulteration can also lead to social unrest, as people become angry and frustrated with a lack of food safety (Hika, 2020; Solomon, 2015). It can also result in nutrient deficiencies, which can negatively impact growth, development, and immunity, and increase disease vulnerability (Majed et al., 2016). Adulteration of food has an adverse impact on producers and farmers, consumers, enterprises, and government (Ayza & Belete, 2015).

Therefore, designing simple and feasible detection methods for food adulteration is essential to ensure the safety of food products. Adulteration can be detected using a variety of techniques depending on the type of adulteration being detected. Several techniques can be used to detect adulteration in food. These include analytical, physical, chemical, and latest DNA‐based molecular techniques (Bansal et al., 2017; Hong et al., 2017). Understanding common types of foods to be adulterated, adulterants, and health implications of the different adulterants is very important to the consumer (Anita & Neetu, 2013). Therefore, this review briefly discusses the adulteration of selected food items, their health impacts, and analytical techniques used to detect food adulteration.

## 2 METHODOLOGY OF THE REVIEWS

A systematic literature search was considered an essential part of the review process. Hence, an extensive literature search was conducted to collect relevant scientific evidence using various sources, such as PubMed, Google Scholar, Science Direct, Scopus, and Web of Science. Keywords such as adulteration, food adulteration, analytical approach, detection method, and health effects were used to search for relevant articles. Only terms related to this topic were selected for this review.

### 2.1 Concept of food adulteration

Food adulteration is generally defined as the addition or subtraction of any ingredient from food in a way that affects the normal composition and value of the food. It was first studied in 1820 by the German chemist Frederick Accum, who discovered many toxic metals in food and drink items (Bansal et al., 2017; Choudhary et al., 2020; Ghimire, 2016). According to Ayza and Yilma (2014), food adulterants are chemical ingredients that should not be enclosed inside our food or drink and maybe deliberately added to more costly materials to increase their size and decrease manufacturing costs.

According to the World Health Organization (WHO), food adulteration can be explained by the intentional addition of prohibited substances to partially or completely replace healthy ingredients or falsely produced fresh products (WHO, 2017). Adulteration is defined by the Ethiopian Food and Drug Administration (EFDA) as the addition of any foreign substance or ingredient to food or replacing the content of the product with another substance in order to increase the mass or weight of a product or decrease its quality, or enhance its value (EFDA, 2019). Similarly, the US Food and Drug Administration (FDA) considers adulterated food if a substance dangerous to health is added; a cheaper or lower‐quality product is added to the food, a valuable ingredient is removed from the basic food, substandard food quality, or any substance added to increase mass or weight to make it appear more valuable (Bansal et al., 2017). This practice can take place at any of the stages in the supply chain of food preparation either for marketable profit or due to carelessness and lack of proper hygienic conditions of processing, storing, transportation, and marketing (Nasreen & Ahmed, 2014).

The main reason for food adulteration is financial gain. Food businesses often add cheaper and lower quality ingredients to food products to increase their profit margin. For example, adding water to milk, sawdust to ground spices, or adding synthetic colors to fruits and vegetables are common forms of food adulteration (Choudhary et al., 2020). Another reason for food adulteration is lack of proper ingredients regulation and enforcement of food safety standards. In many countries food laws are weak and food regulators are understaffed, poorly trained, or corrupt. This creates an environment where dishonest food businesses can thrive and engage in food adulteration with impunity (Momtaz et al., 2023).

### 2.2 Types of food adulteration

Food adulteration is classified into three types: intentional, incidental, and metallic (FSSAI, 2012).

#### 2.2.1 Intentional adulteration

Intentional adulteration of foodstuffs refers to the addition of substandard substances that have the same properties as the foodstuffs with which they are mixed. Therefore, it is difficult to isolate these harmful factors. Adulterants can be physical or biological in nature. Such adulteration is practiced by unscrupulous producers and traders to intentionally adulterate various foods to increase the level of essential nutrients after reducing a certain amount to increase the economic benefits (Ayza & Yilma, 2014; El‐Loly et al., 2013; Faraz et al., 2013; Narayan, 2014; Tomar & Alka, 2022). This is the riskiest form of adulteration because nutrients are reduced and foreign substances are introduced into food by business‐oriented people who have simply forgotten the humanity behind the money‐making mentality (Awasthi et al., 2014).

#### 2.2.2 Incidental adulteration

Accidental food adulteration is usually caused by carelessness, negligence, ignorance, or lack of proper facilities and hygiene in food processing from farm to table (Bansal et al., 2017). In unintentional food adulteration, producers or traders/retailers are not in a position to add different adulterants, but the ways in which the products are produced, handled, passed, processed, stored, transported, and marketed may be the places where they are adulterated because any substance without its original is extraneous to the products (Ayza & Belete, 2015; Ayza & Yilma, 2014). These adulterants are grouped under the dropping of rodents and larvae into food, pesticide residues, and preservatives (Narayan, 2014). The most common adulterant residues found in plant products are pesticides and DDT (Pandit et al., 2002). The maximum accepted limit for DDT is 3 ppm (Nagvanshi, 2015).

#### 2.2.3 Metallic adulteration

**FIGURE 1:**
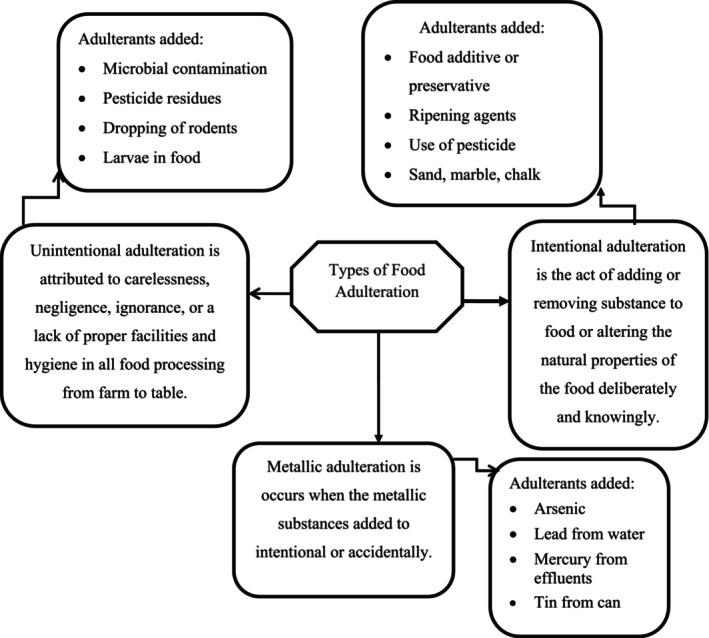
Types of food adulteration.

Metal adulteration is another type of food adulteration. This occurs when metallic substances are intentionally or accidentally added (Bansal et al., 2017). Metal contamination or adulteration includes arsenic from pesticides, lead from water, mercury from chemical effluents, and cans (Thangaraju et al., 2021). Figure 1 summarizes the types of food adulteration and examples of substances added (Pardeshi, 2019a, 2019b).

## 3 SELECTED FOOD ITEMS ADULTERATION AND THEIR IMPACT ON PUBLIC HEALTH

### 3.1 Adulteration of selected food items

#### 3.1.1 Milk and milk products

Milk is defined as the normal milk secretion obtained by milking a completely healthy dairy animal without addition or extraction, and intended for consumption as liquid milk or further processing (Moosavy et al., 2019). Milk is a so‐called “perfect food, because it contains many nutrients. It is the best source of proteins, fats, carbohydrates, vitamins, and minerals for babies and adults” (Azad & Ahmed, 2016). Milk is consumed by large people worldwide, owing to its nutritional value. This has increased the global demand for milk, which could not be met due to reduced production and supply (Salih & Yang, 2017). Thus, the gap between the demand and supply of milk has created a fraud called milk adulteration (Nagraik et al., 2021; Reddy et al., 2017). Following the discovery of melamine contamination in Chinese newborn milk products (Reddy et al., 2017), which resulted in illness of 294,000 children, 50,000 hospitalizations, and 6 deaths, milk adulteration has become a global concern (Saeed et al., 2021).

According to Faraz et al. (2013), milk and its products are intentionally adulterated in order to increase the level of essential nutrients after reduction of a given amount and/or to mislead consumers to increase their profit margin by several chemicals, such as urea, starch, flour, cane sugar, vegetable oils, and detergents. Economically motivated adulteration has resulted in up to 50% butter spoilage (Gemechu et al., 2021). Vanaspati, anatta, and oleomargarine are adulterants found in ghee (Tomar & Alka, 2022).

#### 3.1.2 Fats and oils

Edible oils are both economically and nutritionally important. They provide essential nutrients for human health as they are an important source of mono‐ and polyunsaturated fats (Huq et al., 2022). In our daily diet, fat and oil are commonly used as resources for cooking, frying, and food preparation (Azadmard‐Damirchi & Torbati, 2015). The authenticity of oils and fats has been a major problem since ancient times (Huq et al., 2022). Due to their greater demand for national and international markets, adulteration in high‐priced oil with low‐priced oil is a major issue (Tan et al., 2021; Yadav, 2018).

There are two major adulterations in edible oils and fats: (1) admixing cold press oil with refined oil and (2) replacing more expensive oils and fats with cheaper oils (Huq et al., 2022; Jeep, 2002). The first adulteration of fat and oil begins with admixing cold press oil with refined oil, which is used in the adulteration of cold press oil. During the refining process, trans‐fatty acids and steradienes, which are generally absent in cold‐press oil, are formed. Trans‐fatty acids are not essential, and they do not promote good health. Consumption of trans‐fatty acids increases the risk of coronary heart disease (Salah & Nofal, 2021).

The use of less expensive oil in place of more expensive oil is another way of adulteration. For instance, olive oil is adulterated with vegetable oils containing canola, rapeseed, and mustard to obtain additional profits, which has become a major problem (Azadmard‐Damirchi & Torbati, 2015). Due to the significant price difference, replacing high‐quality oil with inexpensive low‐quality oil is the main technique used to adulterate vegetable oil (Yang et al., 2022). Christopoulou et al. (2004) reported that olive oil is adulterated with sunflower, soybean, cottonseed, corn, walnut, sesame, safflower, and canola oils. Ghee is mostly adulterated with vegetable oils and animal fats (Ahmed et al., 2020). Synthetic colors and flavors are added to other fats to make them look like ghee (Choudhary et al., 2020).

#### 3.1.3 Food grain adulteration

Food grain adulteration involves mixing sand or gravel to increase the weight of the food grains. The cereal grains and pulses were mixed with plastic beads that resembled grains in color and size. Water is also sprayed on grains to increase their weight (Choudhary et al., 2020). Wheat is often adulterated with ergots, which are fungi that contain poisonous substances that are harmful to health.

#### 3.1.4 Fruits and vegetables

Vegetables of different colors and textures are often dyed with different colors and substances. These vegetables are mostly adulterated with malachite green, a chemical dye known to cause cancer. Common adulterants in fruits and vegetables include oxytocin, saccharin, wax, calcium carbide, and copper sulfate. Calcium carbide is used to quickly ripen green fruits artificially, such as bananas, mangoes, guavas, papayas, tomatoes, and pineapple (Okeke et al., 2022). Calcium carbide contains toxic materials such as arsenic and phosphorus which causes blindness and skin irritation (Sharma & Paradakar, 2010). It had a greater concentration of arsenic, which can cause lung, liver, and kidney cancer (Dongre et al., 2020; Mursalat et al., 2013). Because they are injected with chemicals and sprayed with hazardous pesticides and chemicals, market‐sold fruits and vegetables are not free from adulteration (Goyal et al., 2022).

#### 3.1.5 Species

Spice is obtained from the dried part of a plant, which contains seeds, fruits, roots, bark, buds, or other vegetation in addition to leaves (Pantola & Agarwa, 2022). Spices are of great economic importance because they have many different functions such as taste, color, smell, preservatives, and medicinal properties (Negi et al., 2021). They are usually found in powder or powdered form, making them a prime target for adulteration. Spices such as chili, coriander, turmeric, anise, black pepper, and asafetida are all adulterated. The most common adulterants of spices are brick powder, artificial color, chalk powder, papaya seeds, horse manure, lead chromate, etc (Negi et al., 2021). According to Negi et al. (2021), about 7% of spice lots were rejected due to accidental adulteration.

#### 3.1.6 Honey, tea, and coffee

Honey, tea, and coffee are the most likely food ingredients to be targeted for intentional or financially motivated food adulteration. Honey is a healthy, nutritious, and healing bee product and is consumed by the majority of the world's population. It is widely used as a sweetener in the food industry and is found in many processed foods (Cozzolino et al., 2011). Its market value is much higher than other commonly used sweeteners such as refined cane sugar, beet sugar, corn syrup, maple sugar, and high‐fructose corn syrup (Ruiz‐Matute et al., 2010). As a result, honey is adulterated with artificial sweeteners. A review conducted by Teferi (2020) showed that honey in Ethiopia is usually adulterated with sugar, ripe bananas, water, molasses, sugar syrup, corn and/or wheat flour syrup, and sweet potato flour or syrup. Adulteration of honey not only affects national and global market opportunities for the product but can also cause nutritional and health damage to consumers (Gemeda et al., 2020).

Tea and coffee are the most popular beverages worldwide. They play an integral role in our lives; it is so common that millions of people around the world incorporate them into their daily lives (Sharma et al., 2020). Therefore, tea and coffee are very prone to adulteration. The tea is adulterated with foreign leaves or exhausted tea leaves and artificially colored sawdust (Bansal et al., 2017). Coffee is adulterated with tamarind seeds and chicory powder (to add bulk and color) (Lakshmi, 2012).

### 3.2 Impacts of food adulteration on public health

Food adulteration has been linked to diarrhea, abdominal pain, nausea, vomiting, visual disturbances, headache, cancer, anemia, insomnia, muscle paralysis, brain damage, stomach disorders, dizziness, joint pain, liver disease, dropsy, gastrointestinal problems, breathing difficulties, swelling, cardiac arrest, glaucoma, and glaucosis, according to several authors (Tomar & Alka, 2022; Anita & Neetu, 2013, Faraz et al. 2013). Melamine is a nitrogen‐rich compound that is added to milk to increase the natural protein content (Nagraik et al., 2021). Consumption of melamine with food products causes renal failure, kidney stones, and infection in the urinary tract (Rahman et al., 2015).

In China, the use of melamine in baby formula killed 6 children and sickened 300,000 babies in 2008 (Sharma & Paradakar, 2010). According to Nagvanshi (2015), oil adulteration may result in gall bladder cancer, epidemic dropsy, glaucoma, loss of eyesight, paralysis, liver damage, and cardiac arrest. In Spain, “olive oil syndrome” killed over 600 people because nonedible rapeseed oil was sold as edible (Salah & Nofal, 2021). Consumption of fruits adulterated with ripening agents (like calcium carbide) has been confirmed to be carcinogenic to the human body (Goonatilake, 2008). People consuming food preserved with formaldehyde have fallen victim to disturbances in the nervous system, kidneys, liver, and lungs (Momtaz et al., 2023; Nowshad et al., 2018)

In 2013, methanol poisoning affected 69 patients, 8 of whom died in Iran (Momtaz et al., 2023). In 2018, methanol poisoning caused massive hemorrhagic cerebral infarction or multiple organ failure in Malaysia, affecting 90 people and causing 6 deaths (Visciano & Schirone, 2021). Some known cases include dioxins in pork meat in 2008; milk, fat, and urea treated with detergent in 2012; and beef products treated with horse meat in 2013 (Kamruzzaman, 2016). Worst of all, some adulterated foods even cause cancer, which is the most life‐threatening disease (Singh et al., 2021). Irrespective of the type of adulteration, prolonged consumption of adulterated food is very harmful to the human body (Everstine et al., 2013; Srivastava, 2015). Table 1 summarizes selected food items, adulterants, and their impacts on public health.

**TABLE 1:** Selected food items, adulterants, and their impacts on public health.

Selected foods	Adulterants	Their impacts on public health
Milk	Water, starch, urea, and extraction of fat	Digestive system disorder
Sugar	Chalk powder	Stomach infections
Tea	Artificial pigments/dye and iron fillings	Liver disorders and cancer
Coffee powder	Tamarind and date seed powder and sawdust	Diarrhea
Salt	White powder, stone, and raw	Stomach disorder
Chili powder	Artificial colors, brick powder, and Sudan dye	Blood and lung cancer
Turmeric	Lead chromate, sawdust, and metal yellow	Carcinogenic
Mustard seed	Seeds of prickly poppy Argemone	Epidemic dropsy and glaucoma
Black pepper	Dried papaya seed	Cardiac arrest and injurious to health
Food grains, pulses, etc.	Sand, stone, marble chips, and filth	Damage digestive tract
Butter	Margarine and starch	Food poisoning
Honey	Fructose syrup/cane sugar	Stomach disorder
Sweets juices	Coal tar dye/metal yellow	Cancer
Green chilies, green peas, and other vegetables	Malachite green	Cancer
Vegetable oil	Argemone mineral oil	Heart disease, skin infection, and cancer
Ghee	Ghee essence, vanaspati, sweet potato, mashed potato, and starch	Cancer and acute renal failure
Carbonic drinks	Aluminum leaves	Asthma and lung disorder
Rice and wheat	Mud grits, soapstone bits, sand, and Ergot	Cancer and genetic mutations harm the human reproductive system
Seafood	Mercury and arsenic	Stomach and brain disorder
Jaggery	Washing soda and chalk powder	Vomiting and diarrhea

*Note*: Lakshmi (2012); Bansal et al. (2017)

## 4 METHOD FOR DETECTION OF FOOD ADULTERATIONS

**FIGURE 2:**
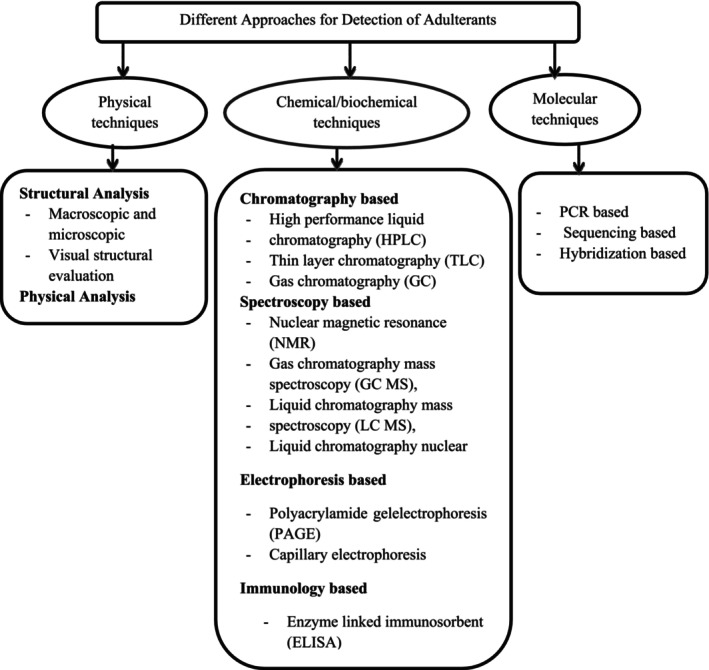
Various analytical approaches to detect adulterants. Source: (Bansal et al., 2017).

Food item adulteration can be detected by different techniques based on the type of adulterant to be detected. These techniques comprise physical, chemical/biochemical, and most current DNA‐based molecular techniques (Figure 2).

### 4.1 Physical methods

Physical methods of adulteration detection including microscopic and macroscopic visual structural assessments, as well as analysis of physical parameters such as morphological characteristics, structure, solubility, and bulk density, have been designed but these methods do not guarantee qualitative adulterant detection (Bansal et al., 2017; Dhanya & Sasikumar, 2010). According to Mangal et al. (2014), visual structural analysis by macroscopic and microscopic methods is very valuable for identifying microbes as harmful types, especially fungi. Microscopic examination easily detects excess starch in powdered spices such as cumin, coriander, chili, and cassava (FSSAI, 2012). Adulteration of honey with cane sugar and cane sugar products can be detected with an optical microscope (Kerkvliet & Meijer, 2000). For impurities inherent in cereal foods such as dust, stones, straw, weed seeds, damaged fruit, shriveled fruit, insects, rodent hair and secretions, etc., adulteration can be detected by sampling a small amount on a glass plate and visually examining impurities because clean foods do not contain such impurities (Banti, 2020).

### 4.2 Chemical/biochemical methods

Chemical/biochemical techniques used for food item adulteration detection can be categorized into four groups:

1. Chromatography based
2. Spectroscopy based
3. Immunology based
4. Electrophoresis based (Bansal et al., 2017).

#### 4.2.1 Chromatography‐based techniques

Chromatography is a sensitive technique used to separate, identify, and purify small molecules in a mixture, such as fatty acids, carbohydrates, and amino acids, for qualitative and quantitative analyses (Salah & Nofal, 2021). Chromatographic methods are the most common priority for assessing the authenticity of most foods. This is partly because techniques such as chromatography can be used both to detect adulterants and as a motive to determine authenticity (Pastor et al., 2019). Modern analytical methods like high‐performance liquid chromatography, gas chromatography–mass spectrometry, etc. can accurately identify the types and concentrations of different types of food degrading agents (He et al., 2021). According to Bansal et al. (2017), gas chromatography is often used for the separation of different types of unseparated products, for adulteration detection, and for the authentication and identification of organic substances. According to Nagraik et al. (2021), high‐performance liquid chromatography coupled with other detection systems has been regularly used for the detection of various milk adulterants. Chromatography combined with mass spectroscopy (MS) and Fourier transform infrared spectroscopy (FTIR) has been widely used for the detection of harmful materials in food (Nogueira & Nascimento, 1999).

#### 4.2.2 Spectroscopy‐based techniques

Bacon et al. (2004) defined spectroscopy as the study of the absorption, transmission, and emission of light and other radiations of matter and their dependence on the wavelength of the radiation. Spectroscopic techniques like near‐infrared (NIR), mid‐infrared (MIR), and Raman spectroscopy have been widely and successfully used as sensitive and fast analytical techniques for detecting food adulteration (Lohumi et al., 2015). These techniques have the advantage of being nondestructive and having a relatively low analysis cost (Nagraik et al., 2021). The Fourier transform infrared (FTIR) based on mid‐infrared (MIR) spectroscopy is one of the most commonly used spectroscopic technique for food fraud currently used by both industry and government laboratories (Valand et al., 2020).

By using detailed spectral inspection, Fourier transform infrared spectroscopy (FTIR) can distinguish between adulterated and unadulterated samples (Ozen & Mauer, 2002). It has been used to detect food adulteration in high‐quality foods such as virgin olive oil (Georgouli et al., 2017) and honey (Gok et al., 2015; Rios‐Corripio et al., 2011; Siddiqui et al., 2017). The presence of palm kernel olein as an adulterant agent in coconut oil can be successfully detected by Fourier transform infrared spectroscopy (Yadav, 2018). According to Nicolaou et al. (2010), Fourier transforms infrared spectroscopy (FTIR) has a high potential for detecting milk adulterants. This enables rapid and simultaneous detection of adulteration and prediction of quality indicators, regardless of the type of milk (Sen et al., 2021). Spectroscopic techniques combined with chromatography have great potential for adulteration detection (Ruiz‐Matute et al., 2007); for example, GC–MS showed potential to detect honey adulteration using commercial syrups. Fourier transform infrared spectroscopy (FTIR) provides a detailed spectral fingerprint to distinguish spice adulterants (Negi et al., 2021). Sunflower and corn oils are common adulterants of olive oil and can be detected by Fourier transform infrared spectroscopy (FTIR) combined with chemometrics (Banerjee et al., 2017).

#### 4.2.3 Immunology‐based techniques

Enzyme‐linked immunosorbent assay (ELISA) is the most widely used immunoassay in adulterant detection and has the advantages of high sensitivity, ease of use, reliability, low cost, and rapid application over other techniques (Bottero et al., 2002; Popelka et al., 2002; Negi et al., 2021). As reported by Castro et al. (1992), this indirect enzyme‐linked immunosorbent assay was developed for the detection and quantification of bovine milk adulteration in goat's milk. It has been noted that an enzyme‐linked immunosorbent assay can be successfully functional in the detection of cow milk adulteration of sheep, goat, and buffalo milk (Hurley et al., 2004; Nagraik et al., 2021; Xue et al., 2010). The enzyme‐linked immunosorbent assay (ELISA) is the most common immunological technique used for the detection of foreign proteins in milk and other foods (Nagraik et al., 2021). This assay can be employed as one of the techniques for the detection of melamine in milk (Garber, 2008). It was recommended that enzyme‐linked immunosorbent assays could be used as the repetitive inspection apparatus for milk (Sheikha, 2019).

#### 4.2.4 Electrophoresis‐based techniques

The electrophoresis technique has also been utilized for the detection of food adulteration. This technique works on the separation of the charged molecule under the applied electric field (Negi et al., 2021). Electrophoresis is an efficient means to detect the purity of a compound and adulteration (Swetha et al., 2017). Among the electrophoresis methods, capillary electrophoresis has shown the capability to detect numerous adulterants from food samples, as capillary zone electrophoresis has been utilized to determine the adulteration of cow milk in goat milk products and adulteration in basmati rice (Cartoni et al., 1999; Vemireddy et al., 2007). Another electrophoresis technique that has the potential for adulterant detection is urea‐PAGE, which has revealed the ability to detect adulteration of milk and in particular the species origin of milk (Bansal et al., 2017; Nagraik et al., 2021).

#### 4.2.5 Molecular‐ or DNA‐based techniques

According to Bansal et al. (2017), DNA‐based molecular is a more powerful technique for the detection of adulterants in traded commodities of plant origin, particularly when the adulterants are biological substances. It can easily discriminate adulterants from food items if both the adulterant and food have a physical resemblance. For utilizing DNA‐based molecular techniques, three approaches are used: PCR based, sequencing based, and hybridization based (Dhanya & Sasikumar, 2010). PCR‐based techniques are simple, sensitive, specific, and low cost. It has a high potential for adulterant detection and authentication of commodities (Bansal et al., 2017). DNA barcoding and restriction fragment length polymorphism (RFLP) are the two approaches that have been confirmed to be very useful in utilizing PCR (Chandrika et al., 2010). PCR is the principal detection method used for the food categories of meat and meat products, fish and seafood, and milk and milk products (Hong et al., 2017).

DNA fingerprinting techniques based on PCR include random amplified polymorphic DNA (RAPD), arbitrarily primed PCR (AP‐PCR), DNA amplification fingerprinting (DAF), inter simple sequence repeat (ISSR), PCR restrict fragment length polymorphism (PCR‐RFLP), amplified fragment size polymorphism (AFLP), directed amplification of minisatellite region DNA (DAMD), sequence characterized amplified areas (SCAR), amplification refractory mutation system (ARMS), and simple sequence repeat (SSR) analysis (Dhanya & Sasikumar, 2010; Bansal et al., 2017).

According to Carles et al. (2005), the hybridization method is used for the detection of adulterants based on small changes in nucleotide strands relative to recognized DNA sequences, and detection can be performed from a variety of possible species at the same time. Although molecular techniques like sequencing and hybridization‐based total approaches are irresistible for biological adulterant detection, prior sequence knowledge is required for designing primers for the amplification of specific sequences (Bansal et al., 2017; Lockley & Bardsley, 2000). The advantages and disadvantages of various methods used for the detection of adulteration in food items are presented in Table 2.

**TABLE 2:** Advantages and disadvantages of food adulteration detection techniques.

Techniques	Advantages	Disadvantages
Physical methods (including microscopic and macroscopic methods)	Very useful for microbial detection, particularly in the case of fungi It is easy to detect extraneous starch and cane sugar used for spices, powder, and honey adulteration, respectively	It does not guarantee the detection of qualitative adulterants. It is not promising to detect the majority of adulterants. It is not a low‐cost routine technique because it necessitates meticulous sample preparation
Chemical and biochemical methods Chromatographic‐based methods (including HPLC and GC)	High resolution High sensitivity Tolerable cost Wide application (quality control, characterization of food products, and detecting adulteration)	Labor intensive Cannot provide quantitative data Often requires statistical analysis. Small analysis scope Complex sample preparation Large consumption of solvent
Spectroscopic‐based methods	Nondestructive testing High accuracy Rapid detection	Expensive equipment Complex testing data
Immunologic‐based methods (including ELISA)	Less costly Straightforward Sensitive Provide both qualitative and quantitative data	Protein denaturation It is not suitable for highly processed samples. Based on biomarkers
Electrophoretic‐based methods (including capillary electrophoresis and urea‐PAGE)	Simple and easy Sensitive Less costly Robust	Degradation profile of a peptide marker It is necessary to refer to sample preparation. Nonquantitative Low throughput
DNA‐based methods (including PCR, sequencing, and hybridization‐based methods)	High specificity High throughput Highly stable DNA biomarkers Highly sensitive Robust, reproducible, and efficient Adaptable	Technically challenging Current high cost Resource intensive Large consumable requirement Need to develop new bioinformatic algorithms to manage large amounts of data

*Note*: El Sheikha (2019).

## 5 CONCLUSION

This article reviewed selected food items adulteration, their impacts on public health, and detection methods. Food adulteration is the intentional lowering of the quality of purchased foods, either by adding or substituting inferior materials or by removing some valuable ingredients from essential foods. Food adulteration has been dangerous for humanity since the dawn of civilization. It is a growing worldwide problem and consequences can be devastating ranging from public health to economic losses. Adulterants can be found in all food we consume in daily life including milk and milk products, vegetables, oils and fats, spices and condiments, and beverages such as coffee and tea. The lack of proper legislation and its strict enforcement is one of the primary reasons for the rapid increase in food adulteration. Thus, it should be necessary to take robust action against adulteration which may help consumers to live healthy life.

Detection of food adulteration became an essential requirement for ensuring both the quality and safety of foods. Detection methods such as physical, chemical, analytical, and immune‐based techniques are used to identify substances in food to provide unadulterated food to consumers. Adulterated food is often not discovered until it causes health risks. Despite advancements in food safety, many developing countries still lack the necessary analysis techniques to detect food adulteration.

Food adulteration is a complicated issue that must be addressed through a multistakeholder strategy. Consumers being the ultimate users of the food products should be fully aware of the adulteration practices that are prevalent among a large number of manufacturers. Education and awareness campaigns should be conducted to inform about the risk of food adulteration and how to protect themselves, as some sort of awareness in this context may prevent life‐threatening diseases and save the lives of innocent people who become the victims of this hazard. Efforts should be made to improve food safety regulation and enforcement. The prevention of food adulteration involves the involvement of governments, food industries, consumers, and civil society organizations. By working together, we can make sure that the food we consume is of high quality, wholesome, and safe.

## AUTHOR CONTRIBUTIONS

**Abdulmajid Haji:** Writing – review and editing (equal). **Kasahun Desalegn:** Conceptualization (equal); writing – original draft (equal). **Hayat Hassen:** Writing – review and editing (equal).

## FUNDING INFORMATION

There is no funding for this review.

## CONFLICT OF INTEREST STATEMENT

The authors declare no conflicts of interest.

## Data Availability

The data used to support the findings of this study are available from the corresponding author upon request.
